# Tolerant mothers: aggression does not explain solitary living in the bush Karoo rat

**DOI:** 10.1098/rspb.2024.1534

**Published:** 2024-10-02

**Authors:** Lindelani Makuya, Neville Pillay, Siyabonga Patrick Sangweni, Carsten Schradin

**Affiliations:** ^1^ School of Animal, Plant & Environmental Sciences, University of the Witwatersrand, Private Bag 3, Johannesburg WITS 2050, South Africa; ^2^ IPHC, UNISTRA, CNRS, 23 rue du Loess, Strasbourg 67200, France

**Keywords:** solitary living, social system, aggression, social tolerance, kin neighbours, natal dispersal

## Abstract

Many mammal species are thought to adopt solitary living owing to mothers becoming intolerant of adult offspring and the occurrence of social intolerance between adults. However, field studies on how solitary mammals interact are rare. Here we show that solitary living can occur without social intolerance. Over 3 years, we recorded interactions between free-living bush Karoo rats (*Otomys unisulcatus*) and conducted dyadic encounter experiments between kin and non-kin female neighbours, both in a neutral test arena and in field intruder experiments. Social interactions were rare (230/2062 observations), and they were aggressive in only 34% of cases. In dyadic encounters, mothers interacted amicably with young offspring. Aggression between mothers and offspring was almost absent. This mother–offspring relationship remained amicable even after adult offspring had dispersed. Aggression between neighbouring adult females was low in neutral arena tests, independent of kinship and season. However, in the field, females reacted more aggressively towards non-kin than kin intruders, especially during the breeding season. Tolerance between mothers and adult offspring indicates that aggression is not the mechanism leading to dispersal and solitary living. We found a solitary social system characterized by social tolerance, suggesting that dispersal and lack of social attraction rather than aggression can lead to solitary living.

## 1. Introduction

To understand the diversity of social systems, many studies have focused on pair- and group-living species, assuming solitary living to be the ancestral state that does not require any explanation for its occurrence [1–3]. Solitary, pair and group living refer to the social organization, describing the composition of social units [4]. Social organization is one of four components of the social system, which further includes the care system, mating system and social structure [4,5]. Of all the different forms of social organization, solitary living is the most understudied [2].

Previous studies regarded solitary living as the ancestral default form of social organization in mammals [1]. However, recent comparative studies have shown that this is often not the case [6,7]. These studies showed that pair-living was most likely the ancestral form of social organization in artiodactyls [8] and primates [7], and possibly also in marsupials [9] and Eulipotyphla [10], indicating that solitary living is often a derived state [2]. However, we know little about the mechanisms leading to solitary living in mammals [11]. To understand the mechanisms of group living, we must also understand the alternative, which is solitary living [3].

It is generally assumed that mammals live solitarily owing to aggression. This opinion is based on studies of the European hamster (*Cricetus cricetus*) in the 1950s [12]. In this species, mothers become intolerant of their offspring when they reach puberty and adults are highly intolerant of each other [12]. The assumption that social intolerance is the main reason for solitary living in mammals was also based on standardized laboratory experiments on solitary rodents that examined the proximate mechanisms of aggression [13–15]. However, whether this is always the mechanism leading to solitary living under natural conditions is unknown. As an alternative to social intolerance and aggression, a lack of social attraction combined with a motivation to disperse when reaching sexual maturity could lead to solitary living. In solitary mustelids, for example, individuals commonly meet in a non-aggressive context [16]. Social interactions in nature have been studied in a few solitary-living species such as the puma (*Puma concolor*) [17] and the giant kangaroo rat (*Dipodomys ingens*) [18]. While social interactions in these solitary species were rare, these studies highlight the lack of aggressive interactions when individuals met. More field studies are needed to understand the mechanisms that cause solitary living.

Kin selection has been used to explain interactions in group-living species, but it might also influence solitary species, predicting close kin to be more tolerant towards each other [19]. Solitary species can have complex social structures in which individuals interact in a non-random way [11]. They often display kinship-determined spatial patterns where kin live close to each other and share part of their range and engage in non-aggressive interactions when they meet [17,18]. The kinship patterns in these solitary species are driven by philopatry, a behaviour usually displayed by group-living species, with individuals dispersing only short distances and forming kin clusters [20]. For example, the social structure of the giant kangaroo rat is formed by female kin neighbours, with shorter distances between neighbours leading to increased social interactions [18]. The frequency of social interactions was positively related to population density, indicating an influence of season on the social and spatial structure [21]. Amicable interactions at territory boundaries are not a contradiction to the theoretical assumption that aggression is a main driver of solitary living, but could simply be owing to the 'dear enemy' phenomenon, where individuals are tolerant of known neighbours as long as they do not cross territory boundaries [22]. To test whether solitary living can arise without aggression, we need to measure aggression inside territories and at nesting sites. Field experiments are needed to test the level of aggression in solitary species, and whether close kin are more tolerant of each other than non-kin.

Our aim was to test the general assumption that aggression and social intolerance are the mechanisms leading to solitary living in our study species, the bush Karoo rat (*Otomys unisulcatus*) from South Africa. In particular, if aggression is the main mechanism leading to natal dispersal and solitary living, we predicted (i) that the behaviour of mothers changes towards their offspring as the offspring become older, with mothers showing more aggression towards dispersed adult offspring than non-dispersed juvenile offspring. Because our study species has a kin-based spatial structure with significant overlap of home ranges between close kin [23], and because kinship has been largely ignored in interpreting social systems of solitary species, we further predicted (ii) that individuals would show higher levels of aggression towards non-kin neighbours than towards kin neighbours. To test these predictions, we conducted more than 2000 focal animal observations over a period of 3 years, and carried out additional tests in a neutral presentation arena in a field laboratory and field experiments where we presented kin and non-kin neighbours at the nesting sites of resident females.

## 2. Material and methods

### (a) Study site

The study was conducted in the Goegap Nature Reserve, in the Northern Cape, South Africa. The study site is located in the Succulent Karoo biome [24]. The climate is semi-arid, with temperatures falling below 0°C in winter and exceeding 40°C in summer [25]. Mean precipitation at the field site is 160 mm per annum. Seasons are divided into the hot dry non-breeding season (December to May) and the cold wet breeding season (June to November).

### (b) Study species

The 100 g bush Karoo rat offers a model to study solitary living because it is diurnal, occupies an open habitat, has small home ranges (0.06 ± 0.04 ha in the dry, non-breeding season and 0.04 ± 0.03 ha in the wet, breeding season) [23] and easily habituates to the presence of observers. It inhabits the semi-arid regions of South Africa including the Succulent Karoo (less than 200 mm of rain per annum), which is one of the world’s most important biodiversity hotspots [26]. The bush Karoo rat builds stick-lodges inside shrubs, which offer a favourable microclimate with high humidity and mild temperatures as a buffer against the harsh outside environment that is characterized by unpredictable rain in cold winters, and long, hot summer dry seasons [27–29]. The bush Karoo rat is a central place forager, foraging around its stick lodge and taking food back to the lodge, where it can be easily observed and where experiments can be conducted. In the Succulent Karoo, around 95% of the rats are solitary, although a few small groups of 2–3 closely related females occur [23]. They have a kin-based spatial structure, with female kin living close to each other, and home ranges of close kin overlapping more with each other than with non-kin [23]. Young male bush Karoo rats behave similarly to females at the beginning of the dry season and stay in an area close to their natal lodge. However, they disperse in winter when food availability increases, just before the beginning of the breeding season. Adult males roam over very large areas and during the breeding season there are no male neighbours because there are no resident males.

### (c) Sampling regime

#### (i) Marking and trapping

Trapping was conducted at lodges that showed signs of being occupied (fresh faeces, active runways, rats observed). The field site was divided into six areas, with 1–2 areas trapped simultaneously by two research teams. All lodges within one area were trapped for three consecutive days before moving on to the next area. Traps were set in the morning before sunrise, checked after 45 and 90 min and then un-set during the hottest times of the day. In the afternoon, traps were set at 45 min before sundown, checked after sundown and then un-set during the night. We used a combination of foldable Sherman traps (https://shermantraps.com/) and locally produced heavy metal traps of the Sherman style. Traps were baited with a combination of bran flakes, salt and sunflower oil and re-baited each morning and afternoon. Traps were arranged around the entrances to the lodges and along runways. We recorded the body weights of all individuals to the nearest 0.1 g, as well as their sex, reproductive status and lodge number. We marked individuals with single, metal-band ear tags that had a unique reference number (National Band and Tag Co., Newport, KY, USA) [30]. To aid in visual identification during observations, individuals were marked with non-toxic hair dye (Inecto Rapido, Pinetown, South Africa), in combinations (females: head and chest/sides/back; males: hindquarters and chest/sides/back). Age was estimated from body mass at first capture, using a species-specific growth curve [31] validated for our field data. The bush Karoo rats were classified according to their age, with individuals up to two weeks old and weighing less than 30 g scored as pups (weaning is at 14 days; [31]), those between 2 and 6 weeks old and weighing 30 and 70 g as juveniles and those older than six weeks and weighing more than 70 g—when both sexes can start reproduction—as adults [31].

#### (ii) Determining dispersal

The onset of dispersal was determined for the rats used during the dyadic encounter tests (explained in §1c(iv)). We determined these occurrences from the time when the rat was trapped consistently over a period of more than four weeks at a lodge that was not its natal lodge, without the mother being trapped and observed at the same lodge. Using these data, we calculated the age at dispersal.

#### (iii) Focal animal observations in the field

Focal animal observations were conducted from July 2021 to October 2023 for a total of 2062 observations and comprising 246 rats at lodges with identified bush Karoo rats. The observations established (i) which individuals occupied the lodges, and (ii) recorded behaviours, including interactions with conspecifics. The observations were made for 30 min in the morning after sunrise and 30 min in the afternoon before sunset. Observations were made using focal animal sampling and one zero recording for 30 min. We recorded all social behaviours in 1 min intervals. The social behaviours included the following groups of behaviour: (i) amicable (e.g. grooming, body contact); (ii) social investigation (i.e. sniffing); and (iii) aggression (e.g. chasing, fighting).

#### (iv) Dyadic encounter tests

Dyadic encounter experiments were used to assess whether interactions with neighbours were amicable (predicted for close kin) or aggressive (predicted for non-kin). Trapped Bush Karoo rats were transported 100m to a laboratory at the research station and allowed to acclimatize for 10 min before the start of the experiment. The experiments were conducted in a neutral test arena that was constructed from woodchip panels (80 cm × 65 cm × 94 cm) and had a partition down the middle (electronic supplementary material, figure S1). The testing arena was cleaned between encounters using diluted Dettol Antiseptic Liquid and then air dried. All tests were done between 10.ooam and 12.00 noon, and they took place from August 2021 until March 2023, for a total of 143 tests on 52 focal rats. Each rat was tested 2.80 ± 2.2 times on average.

Each bush Karoo rat was introduced into the arena and allowed to settle for 5 min with the partition down. Initially, mothers were denoted as the focal individual and they were tested against their offspring, which were from 1 month and 20 months old (*n* = 80 tests, 3 with male offspring and 77 with female offspring, using 31 focal mother rats). The offspring tested were either still living in their mothers’ lodge, i.e. not dispersed (*n* = 43), or already dispersed (*n* = 36). We attempted to test each mother with the same offspring at different ages, but because some rats were not re-trapped and disappeared, some mothers were tested with different offspring at different ages. Next, we tested adult female focal rats on two different days with an adult female kin neighbour and an adult female non-kin neighbour, respectively (*n* = 63 tests on 41 rats). Half of the rats were first tested with a kin neighbour and the other half with a non-kin neighbour. We defined a neighbour as a rat that occupied a lodge not more than 25 m away from the focal rat. A total of 22 of the focal rats tested with a neighbour were used in the tests with offspring. Because all stimulus animals were direct neighbours, our experimental design controlled for the dear enemy phenomenon, whereby the owner of a territory responds less aggressively to a familiar neighbour than towards a stranger [22]. Presentations lasted for 15 min each. The focal adult individual was heavier than the stimulus female (mean weight difference 25.8 g ± 20.3 g s.d.) because we wanted the focal individual, which we assigned as the owner of the territory, to initiate the encounters and body mass difference was expected to have a positive influence on the initiation of aggression [32]. At the end of each test, bush Karoo rats were returned to their lodge. Focal animal sampling was used to record the frequency of social behaviours as described for focal animal observation above [33]. The behaviours were recorded for 15 min using a webcam. The observer was present in the same room as the animals being tested but was separated from the animals by a black curtain and they monitored the behaviours live on a computer. Tests would have been immediately terminated as soon as individuals started damaging fights (biting and/or standing upright and boxing for more than 2 s) to avoid any injury. However, this was never needed in our study. Videos of the interactions were scored using BORIS (Behavioural Observation Research Interactive Software [34]).

#### (v) Field intruder presentation tests

Since we observed little aggression during the neutral arena tests, we conducted field intruder tests directly at the rats’ lodges. A similar experiment had been done on group-living African ice rats (*Otomys sloggetti robertsi*) from the alpine regions of the southern African Drakensberg and Maluti mountains [35]. We expected higher levels of aggression here owing to the focal rats defending their territory, which was not the case in the neutral arena in the laboratory. Again, we tested whether focal animals are more aggressive towards non-kin than kin neighbours. These tests were done 1–2 years after the dyadic encounter tests in the neutral test arena, and out of the individuals tested in the field, only nine had participated as the focal (*n* = 9)/stimulus (*n* = 6) in the previous experiments. Stimulus individuals were trapped (as described above) and then transferred into a wire mesh cage (30 × 15 cm, 12 cm height; electronic supplementary material, figure S2). This wire mesh cage allowed other bush Karoo rats to see and smell the stimulus animal in the trap, but not make physical contact. The trapped individual was then presented at a neighbouring rat’s lodge (i.e. the focal individual). The focal individual was not caged and its response to the trapped stimulus individual was recorded. The wire cage was positioned within an active runway 30 cm away from the lodge. Observations started when the focal animal was observed outside its lodge and lasted for 15 min thereafter. The maximum duration of the presentation was 45 min; thus, if the focal animal was not seen within the first 30 min, the experiment was terminated. During the 15 min of observations, we recorded aggressive behaviours of the focal animal towards the caged stimulus animal, including charging towards the cage and emitting aggressive 'chit' sounds. We also recorded the latency until the investigation of the stimulus individual and the first aggression, as well as the total time spent at the cage. Thereafter, the stimulus animal was returned to its lodge at which it had been trapped. Each focal individual was tested once with a kin neighbour and once with a non-kin neighbour on separate days. The tests were conducted in the early mornings between 6:00 and 9:00 from January–November 2023. We conducted a total of 54 tests for 26 focal rats, with each rat being tested 2.03 ± 0.87 times on average. For both the dyadic and field encounter tests, a focal rat could be used as a stimulus in another experiment, and a stimulus rat could be used multiple times for different focal rats. In the dyadic tests, 15 rats were used as both focal and stimulus and 12 rats were used in the intruder tests.

#### (vi) Statistical analysis

All statistical analyses were done in R (v. 4.3.1 [36]). We used an ANOVA to analyse factors that influence the frequency of sniffing events, or the time spent in body contact, fighting or grooming, and we included the age of offspring, and season (breeding versus non-breeding) as factors for the dyadic encounter tests between mother and offspring (table 1).

**Table 1.** Hypothesis and associated models tested in the dyadic and field intruder tests. * Indicates that variables were fit as fixed effects separately and as an interaction.

hypothesis	type experiment	model	model type
the behaviour of mothers changes towards their offspring as the offspring become older.	dyadic encounter—mother : offspring	time in body contact = season + age of offspring.	LME
		time fighting = season + age of offspring.	LME
		time grooming = season + age of offspring.	LME
		frequency of sniffing = season + age of offspring.	GLMM– Poisson
bush Karoo rats show higher levels of aggression towards non-kin than towards kin neighbours	dyadic encounter— neighbours	fight = season + relatedness + body size difference + (1|ID-focal).	LME
		body contact = season + relatedness + body size difference + (1|ID-focal).	LME
		groom = season + relatedness + body size difference + (1|ID-focal).	LME
		sniff = season + relatedness + body size difference + (1|ID-focal).	GLMM– poisson
	field intruder test	latency to first aggression = season * relatedness + body size difference + (1|ID-focal).	LME
		latency to approach = season * relatedness + body size difference + (1|ID-focal).	LME
		charging = season * relatedness + body size difference + (1|ID-focal).	GLMM– poisson
		trills (chit) = season * relatedness + body size difference + (1|ID-focal).	LME
		time at cage = season * relatedness + body size difference + (1|ID-focal).	LME

We ran linear mixed models (LME) in *lme4* [37]to investigate factors that influenced the behaviour exhibited by bush Karoo rats during dyadic encounter tests toward neighbours. For affiliative behaviours, we tested the time spent sitting in body contact and time spent grooming. For aggressive behaviours we tested the duration of fights. Finally, for social investigative behaviours, we fit a generalized linear mixed model (GLMM) with a Poisson distribution, and we tested the number of sniffing events displayed. For all of the models, the duration/frequency of the behaviours were fitted in linear models as predictors, and we included season, body size difference and relatedness as fixed effects and the ID of the focal individual as random effect (table 1).

We used both GLMMs and LMEs to analyse factors that influence the behaviour displayed by the focal rat towards a stimulus for the field intruder presentation tests. We tested the latency to approach the cage and to first aggression, and the number of charging events (fitted with a Poisson distribution) and the number of chit sounds, and total time spent at the cage. One model was fit for each behaviour. We included season, body size difference and relatedness as fixed effects. We further included the interaction between season and relatedness as fixed effects. To avoid singularity, we only fitted the ID of the focal rat as a random effect (table 1).

## 3. Results

### (a) Mother–offspring interactions

During 87 of the 2062 field observations sessions, pups were present at the mother’s nest. On 11 occasions, we observed amicable interactions with the mother (grooming and body contact) and no interactions occurred on 76 occasions; we never observed aggression towards the offspring from mother. Juveniles were present on 240 occasions during the field observations and were observed in amicable interactions with the mother on 20 occasions, and only once in an aggressive interaction.

The mean age of all offspring (*n* = 79) used as stimulus animals was 4.13 ± 3.72 s.d. months. The age of non-dispersed rats in the experiments was 3.81 ± 3.25 s.d. months (range 1–14 months, *n* = 43) compared with 5.21 ± 4.01 months (range: 1–20 months, *n* = 36) for dispersed rats. We calculated Akaike information criterion (AIC) values for models including the age of offspring versus dispersed or undispersed. We found that the AIC values for models including the age of offspring as a predictor were higher than for the models that included whether the offspring had dispersed (electronic supplementary material, table S2). However, the summary tables for both these models were very similar (results and conclusions did not change) and we reported the models including the age of offspring in the electronic supplement. Dyadic encounters between mothers and their female offspring were characterized by sniffing and body contact with little grooming and nearly no aggression (electronic supplementary material, table S1). Social interactions were not influenced by season and did not significantly change after offspring dispersed (electronic supplementary material, figure S3, tables S3–S6). Specifically, mothers were not more aggressive towards dispersed offspring than non-dispersed (electronic supplementary material, table S4, figure S4), nor did they decrease the level of body contact (electronic supplementary material, table S6, figure S3).

### (b) Focal animal observation

In 230 of the 2062 observations, conducted between July 2021 and October 2023, two or more adult rats were present at the focal rats’ lodge. Ninety-seven chases occurred in 79 of the 230 observations. Chases occurred more often during the non-breeding season (*t*‐test; *t*_1728_ *= −*2*.*99, *p* < 0.01). The chases occurred between a male and an unrelated female (41%) or an unidentified neighbour (41%), and between related females (18%).

### (c) Dyadic encounter tests (neutral test arena)

We tested whether adult female bush Karoo rats (focal individuals) showed less aggression towards kin neighbours than towards non-kin neighbours (adult females). The weight difference between the individuals was not a significant predictor in any of the models. Adult female bush Karoo rats showed very little aggression towards their neighbours, irrespective of the season (LME; estimate = 0.016, s.e. = 0.013, confidence interval (CI): −0.0098/0.042, *t*_63_ = 1.2, *p* = 0.22) and relatedness (LME; estimate = 0.009, s.e. = 0.015, CI: −0.02/0.04, *t*_62_ = 0.6, *p* = 0.55) (electronic supplementary material, figure S2, table S7). Although they tended to spend more time in body contact with kin than with non-kin, this difference was not significant (LME; estimate = −2.36, s.e. = 1.316, CI: −5.04/0.31, *t*_63_ = −1.79, *p* = 0.08; electronic supplementary material, figure S6 A) and there was also no significant difference between seasons (LME; estimate: 0.57 + s.e. = 1.094, CI: −1.63/2.8, *t*_61_ = 0.52, *p* = 0.60) (electronic supplementary material, table S8). The time spent grooming neighbours was not significantly affected by season (LME; estimate = 0.008, s.e. = 0.019, CI: −0.0295/0.045, *t*_63_ = 0.42, *p* = 0.673) or relatedness (LME; estimate = −0.02, s.e. = 0.022, CI: −0.0645/0.025, *t*_58_ = −0.93, *p* = 0.356) (electronic supplementary material, figure S6 B, table S9). Bush Karoo rats spent a significant amount of time investigating neighbours through sniffing during the breeding season (GLMM; estimate = 0.73, s.e. = 0.27, CI: 0.22/1.29, *t* = 2.697, *p* < 0.01) but this was not affected by relatedness (estimate = 0.54, s.e. = 0.36, CI: −0.17/1.27, *p* = 0.14) (electronic supplementary material, figure S7, table S10).

### (d) Intruder field presentation tests

**Figure 1.**
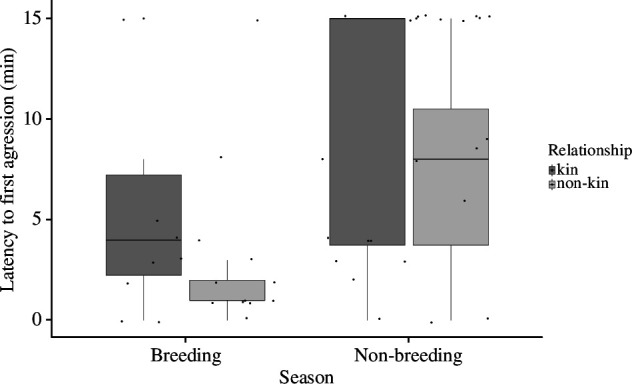
The latency to first aggression by focal females to neighbouring cage-housed female bush Karoo rats during intruder tests. Boxplots show median and first and third quartiles, the whiskers represent the minimum and maximum of the outlier data and points represent individual values.

**Figure 2.**
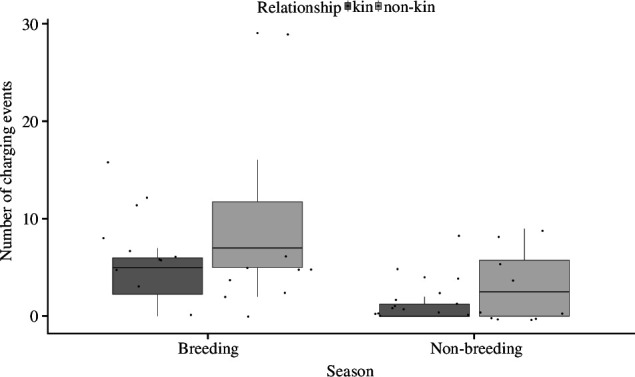
The number of charging events by focal females to neighbouring cage-housed bush Karoo rats during intruder tests. Boxplots show median and first and third quartiles, the whiskers represent the minimum and maximum of the outlier data, and points represent individual values.

**Figure 3.**
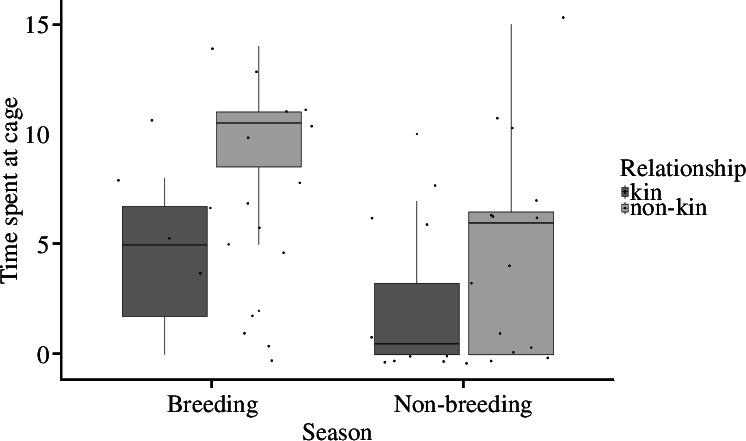
The time spent by focal females at the cage of neighbouring cage-housed bush Karoo rats during intruder tests. Boxplots show median and first and third quartiles, the whiskers represent the minimum and maximum of the outlier data, and points represent individual values.

In both seasons, female bush Karoo rats were mildly aggressive towards non-kin neighbours but were more tolerant of kin neighbours when presented at their lodge. There was no difference in the latency to approach the cage with a kin or non-kin focal neighbour (estimate = −0.98, s.e. = 1.61, CI: −4.27/2.29, *p* = 0.6; electronic supplementary material, figure S8, table S12), indicating that resident bush Karoo rat females investigated the stimulus animals regardless of kinship. However, non-kin females were attacked much faster (LME; *t*_38_ = 2.54, *p *< 0.05, figure 1, electronic supplementary material, table S11), their cages were charged more often (GLMM; estimate = 0.75, s.e. = 0.22, CI: 0.32/1.2, *t* = 3.4, *p *< 0.001; figure 2, electronic supplementary material, table S13) and they produced more trill sounds (electronic supplementary material, figure S8; although this was not significant because a large outlier: LME; estimate = 9.53, s.e. = 18.2, CI: −25.45/44.77, *t =* 1.56, d.f. = 37.59, *p* = 0.61; electronic supplementary material, table S14). Also, focal individuals spent significantly more time at the cage with the non-kin than kin neighbours (LME; estimate = 4.63, s.e = 1.37, CI: 1.76/7.39, *t*_39_ = 4.06, *p* < 0.01, figure 3, electronic supplementary material, table S15). These differences occurred during both the breeding and the non-breeding seasons. However, in the breeding season, female bush Karoo rats showed more aggression towards intruders, regardless of kinship (LME; *t* = −4.32, *p* < 0.001), by quickly attacking (LME; estimate = 4.287, s.e. = 1.82, CI: 0.61/7.95, *t*_43_ = 2.349, *p* = 0.02) and charging at cages (LME; estimate = −1.92, s.e. = 0.44, CI: −2.899/−1.10; figures 1 and 2).

## 4. Discussion

It is typically assumed that individuals of solitary species are intolerant of each other [38]. Here we tested whether female bush Karoo rats live solitarily because of high intra-specific aggression. However, we observed no aggression between mothers and their juvenile or even adult offspring. The mother–offspring relationship did not change when the offspring became older and had dispersed from their mother. In dyadic encounter tests, mother and offspring were found to be in regular body contact, which did not decline as offspring became older. During field observations, mothers interacted rarely with pups and juveniles, but when they did so, it was mainly amicable. Thus, both experimental and field observation data indicate that aggression rarely occurs between mothers and offspring and that aggression does not increase as offspring age and have become solitary. Thus, aggression is unlikely to be the mechanism leading to solitary living. This makes the alternative a likely explanation, i.e. the motivation to disperse when reaching sexual maturity together with an absence of social attraction leads to dispersing individuals settling alone inside an unoccupied lodge.

It is typically assumed that aggression is the main mechanism leading to offspring dispersal and solitary living [39]. The association between aggression and dispersal in which subordinate individuals (juveniles) are driven out by dominant individuals (adults) is well known [39,40]. However, there are equally many studies showing that aggression and dispersal are not always associated with one another [39]. In our study, mothers did not react more aggressively towards their dispersed offspring than towards their offspring still living with them. Female bush Karoo rats reach sexual maturity at six weeks of age [31] and this is thus the earliest age at which they are expected to leave their mother’s lodge (i.e. disperse). Accordingly, females dispersed at two months of age but with a large age variation that needs investigation in the future. For example, food availability, population density and start versus end of breeding season are factors that are expected to influence dispersal. Our study showed the importance of dispersal in becoming solitary, although there was no indication that maternal aggression drives natal dispersal.

Aggressive interactions are often considered as the main underlying reason for solitary living and the reduction of aggression as a first step towards the evolution of sociality [41]. Dyadic encounters between adult neighbouring bush Karoo rats in a neutral arena were characterized by few interactions, independent of kinship, with sniffing being the predominant form of social investigation. Nearly no aggression occurred, not even between unrelated females. However, close kin were more likely to spend time in body contact with each other than non-kin. Thus, while female bush Karoo rats differentiated between kin and non-kin neighbours, aggression was limited in both cases. But there was also no social attraction between adults. Within the same field site, and in similar experiments to ours, group-living striped mice (*Rhabdomis pumilio*) were highly aggressive towards non-kin and interacted amicably with kin [42]. We had expected much more aggression in the solitary bush Karoo rat than in the sociable striped mice, and our ethical clearance protocol included the requirement that experiments would be terminated as soon as damaging fighting started. We expected this to be the case regularly when non-kin met, but we never had to stop the encounter experiments. Therefore, absence of social attraction could be sufficient to lead to solitary living in bush Karoo rats, without the need for social intolerance and aggression.

Interactions between individuals are not random and instead reflect relatedness or familiarity [43]. In field intruder tests, where we expected to find more aggression towards conspecifics, due to the defence of resources [44], aggression was much more common during the breeding than the non-breeding season, and females were much more aggressive towards non-kin than kin. This indicates that female bush Karoo rats can differentiate between kin and non-kin, even when their kin had been living in a different lodge (solitarily) for several months. Kin recognition thus occurs post-dispersal and indicates that mothers remember adult, dispersed offspring. This tells us that remembering kin (the mechanism for kin recognition) persists.

Female territoriality functions to defend resources and offspring [45,46]. This theoretical consideration can explain why we observed nearly no aggression during dyadic encounter tests in a neutral test arena because no resources could be defended, but we did observe some aggression during intruder tests when individuals defended their lodge. Does this indicate defence of resources such as food and shelter, or defence of offspring? More aggression was observed during the breeding season, which is also when more lodges are available owing to low population density at the start of the season, and when food is highly abundant. Thus, our data support the female hypothesis of Wolff and Peterson [44] that territorial aggression is linked more to the defence of offspring (maternal aggression) than the defence of food resources. Reproductive competition can be high between female mammals [47], often leading to female infanticide [48], and is considered as one of the main reasons for solitary living [49]. Several other mammal species show maternal aggression, with females being aggressive at their nests in the breeding season while showing amicable behaviour at communal foraging grounds (reviewed by [44). For example, female Arctic squirrels (*Urocitellus parryii*) and grey squirrels (*Sciurus carolinensis*) defend their territories near their nests but show considerable overlap in foraging areas. This behaviour is not limited to mammals but has been shown also in female social lizards (e.g. White’s skinks, *Egernia whitii*), where female aggression increased during pregnancy and after birth [45]. Aggression in female bush Karoo rats thus rather functions to protect their offspring than to establish a solitary social organization.

## 5. Conclusion

While solitary species have been considered to be generally asocial and aggressive, we have evidence that the solitary bush Karoo rat shows low levels of aggression. Instead, its social system is characterized by social tolerance, which suggests that lack of social attraction after dispersal and not aggression leads to dispersal and solitary living. Female bush Karoo rats were not aggressive to their offspring even after they had dispersed from the maternal lodge and lived solitarily for months. In the solitary bush Karoo rat, maternal aggression is not the mechanism driving offspring to solitary living. Tolerance of conspecifics is a condition for sociality. Therefore, solitary species that can tolerate their conspecifics may provide a model to understand the evolution of sociality in mammals.

## Data Availability

The data are available as supplementary material [51].
